# Annotations

**Published:** 1893-04-22

**Authors:** 


### Annotations.

An analysis of the report of the Leprosy Commission, instituted at the instance of His Royal Highness the Prince of Wales, is published by the *British Medical Journal*. One of the Commissioners, Dr. Beaven Rake, in a letter to the *Journal* says: "To the insinuation of Messrs. Tebb and Lovell that the Leprosy Commission was biassed and unwilling to listen to evidence on the subject of vaccination with reference to leprosy, the report, when it is published, will be a sufficient answer. All available information was sought, not only on the subject of vaccination, but also on every other point connected with leprosy, and all sorts and conditions of men were listened to, from the itinerant Babu, vaunting his secret cures, to the Dominican priest, extolling the merits of the Mattei treatment." The report will give both surprise and satisfaction. In the first place, the allegation of an increase of leprosy in India is not supported by facts. This discovery alone amply justifies the instituting of the Commission. On the subject of heredity, the report of the Commissioners is equally surprising and satisfactory. They announce the conclusion "that leprosy in India cannot be considered a hereditary disease, and they would even venture to say that the evidence which exists is hardly sufficient to establish an inherited predisposition to the disease." A large part of the report is devoted to the burning question of the spread of leprosy by vaccination. On this point, it is stated, with regard to the affirmed "recrudescence of leprosy in India during the last thirty years by means of vaccination," that, in point of fact, there has been no recrudescence at all. There is, moreover, not only no evidence to show that leprosy is spread by vaccination, but none to justify the contention that the natives of India have ever complained that it was spread in that manner. All reasonable men and women will be grateful for the report. For ourselves, we have no intention whatever either of claiming for vaccination more than it is worth, or of insisting upon its compulsory use if it can be shown to be injurious. But we have the strongest possible intention of standing for truth and scientific competency as against the unsupported assertions of the ignorant.

**The Leprosy Commission.**

"In no country is the belief in herbalists so great as in Yorkshire," says the *Yorkshire Evening Post*. Upon which the comment suggests itself that Yorkshire newspapers have a keen eye for the faults of their own county. Is the charge true? It is, and it is not. Yorkshire, in point of size, constitutes about a fourth part of England, and its population is not at all homogenous. The county is divided into three "Ridings," the North, the East, and the West. The North and East Ridings are populated by landed proprietors, farmers, labourers, and retail tradesmen, and the people are generally proud, intelligent, and anything but superstitious. The West Riding is peopled by manufacturers and their factory hands, to which are to be added a great many coal miners. In this latter part of Yorkshire the general education is defective, manners are coarse, dog-fighting and cock-fighting are rampant, and "herbalism" is the chief medical faith. At York the other day, according to the *Post*, an inquest was held upon the body of Mrs. Wormley, who had died of heart disease. Mrs. Wormley had been attended by a herbalist, and he had apparently administered to her nearly nine grains of digitalis every day. Now an ordinary dose of powdered digitalis is half a grain, and a maximum dose, even in the hands of an experienced practitioner, is two grains. The herbalist had, therefore, clearly gone beyond his limit, though there is no evidence to show that his dosing had actually killed the patient. There is really nothing much to be said about the subject. If the public will be foolish, what is to prevent them? Medical diagnosis and the whole medical art is one of the most difficult conceivable of arts. To suppose that it can be taken up and practised by the untaught is one of the stupidest of conceivable absurdities. Vast numbers, even of the taught, never really master the art either of diagnosis or of treatment. If the Yorkshire people, or any other people, have not the brains or the education to understand this, so much the worse for them. We can only express our regret that they are in such an elementary condition of intellectual development. Doctors receive no thanks for trying to save the public from the knaveries of quacks; and so long as the public insists upon being a pigeon it is, perhaps, advisable to allow to be plucked.

**Herbalism in Yorkshire.**

Mr. Josiah Oldfield expresses the opinion in his recent booklet that "John Bull and his boasted roast beef will become 'Consumptive John' through this very consumptive roast beef," if he fails to recognise that tuberculous cattle are dangerous, and that their flesh ought not to be eaten. We congratulate Mr. Oldfield on the skilfulness of his reasoning. He is a special pleader of the very first order; almost as good as the late Mr. Darwin, and much superior to many of his brethren of the bar. But Mr. Josiah Oldfield is "at the bar," and not "on the bench"; and of this fact his monograph contains evidence in almost every line. Medical readers will find both profit and amusement in his pages; non-medical ones will in many cases, very profitably for themselves, be frightened almost "out of their wits." Mr. Oldfield's description of Christmas prize cattle as "bloated hulks of panting fat" is admirable; and as true as it is amusing. That such cattle should be thought "good to eat" is one of those mysteries that, in Dundreary phrase, "no fellow can understand." With Mr. Josiah Oldfield's main contention, that vegetarianism is the hope of the future, we cannot at all agree. But short of this, and, making allowance for forensic exaggeration, we are to a considerable extent in agreement with the writer, and predict for his little work a large measure of well-deserved success. Tuberculosis, in our climate, prevails extensively among cattle, sheep, and pigs. It prevails most of all among milk cows, and especially among those which are kept by milk sellers in the close sheds and stalls of large towns, and fed with food which stimulates the production of milk at the expense of the production of flesh and strength. Mr. Oldfield's facts are really startling, and the present writer knows, of his own knowledge and experience, how generally correct and significant they are. Of all the cattle killed for human food there is a very considerable percentage which are undoubtedly tuberculous; and, what is almost more alarming, of all the cows which are kept for milking there is a still larger proportion that are tuberculous and that are yielding tuberculous milk. Now, tuberculous milk and tuberculous meat may both produce tuberculosis in the human subject; "may," and do produce it. What, then, is to be done? The first thing to be done is to master the facts of the case and to measure their significance. The other things are to agitate and insist upon more competent inspection of meat and milk; and, above all, to cook them both. With adequate inspection and perfect cookery the danger may be reduced to a minimum. But, in addition, there is no reason why we should not all eat far more vegetables than we do and much less meat. The great drawback is that British cooks do not know how tocook vegetables, and British housewives have no capacity for teaching them.

**Vegetarianism and Consumption.**

